# Animal Models of Chronic Pancreatitis

**DOI:** 10.1155/2010/403295

**Published:** 2010-12-14

**Authors:** Makoto Otsuki, Mitsuyoshi Yamamoto, Taizo Yamaguchi

**Affiliations:** ^1^Department of Lifestyle-related Diseases and Dietary Life, Graduate School of Life Science, Kobe Women's University, 2-1 Aoyama, Higashi-Suma, Suma-ku, Kobe 654-8585, Japan; ^2^Department of Gastroenterology and Metabolism, School of Medicine, University of Occupational and Environmental Health Japan, Fukuoko 807-8555, Japan

## Abstract

Animal models for CP in rats can be classified into 2 groups: one is noninvasive or nonsurgical models and the other is invasive or surgical models. Pancreatic injury induced by repetitive injections of supramaximal stimulatory dose of caerulein (Cn) or by intraductal infusion of sodium taurocholate (NaTc) recovered within 14 days, whereas that caused by repetitive injection of arginine or by intraductal infusion of oleic acid was persistent. However, the destroyed acinar tissues were replaced by fatty tissues without fibrosis. Transient stasis of pancreatic fluid flow by 0.01% agarose and minimum injury of the pancreatic duct by 0.1% NaTc solution induced progressive pancreatic injury although one alone is insufficient to cause persistent pancreatic injury. However, the damaged tissue was replaced by fatty tissue without fibrosis. Continuous pancreatic ductal hypertension (PDH) caused diffuse interlobular and intralobular fibrosis closely resembling human CP.

## 1. Introduction

Chronic pancreatitis (CP) is defined as a progressive inflammatory disease of the pancreas, characterized by irreversible morphologic changes and fibrotic replacement of the gland. Epidemiological studies have indicated that chronic alcohol abuse is a major cause of CP. The second common cause of chronic pancreatitis is idiopathic, and the third is recurrent or relapsing pancreatitis due to gallstones [1]. However, the pathophysiology of CP remains poorly defined. Therefore, appropriate therapies are still limited, and prognosis has not improved to date, due mainly to the lack of a satisfactory animal model for CP.

Animal models for CP in rats can be classified into 2 groups; one is noninvasive or nonsurgical models such as chronic ingestion of ethanol and injections of supramaximal stimulatory doses of caerulein (Cn) or toxic substance, and the other is invasive or surgical models such as manipulation of the pancreatic duct or infusion of some toxic substances into the pancreatic duct. Long-term ethanol feeding alone is unable to induce acute or chronic pancreatitis in animals [2, 3]. In addition, CP occurs in only a limited number of heavy drinkers [4–6]. Since these findings suggest that alcohol is a very weak inducer of CP and other factors are necessary for the onset of the disease, we reviewed animal models of CP induced in rats other than chronic ethanol ingestion [7, 8].

## 2. Noninvasive, Nonsurgical Model of Chronic Pancreatitis

### 2.1. Caerulein-Induced Pancreatitis

**Figure 1:**
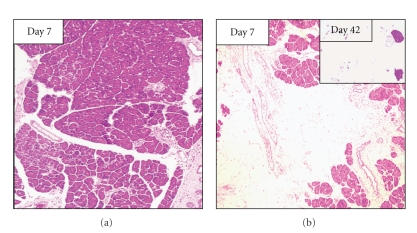
Representative photomicrographs of the pancreas after intraperitoneal (ip) injection of 20 *μ*g/kg body weight of Cn (a) or 500 mg/100 g body weight of arginine (b). (a) Seven days after the last Cn injection (on day 35 after the first Cn injection), the pancreas was recovered and histologically normal. (b) On day 28 after the last ip injection of arginine (on day 40 after the first arginine injection), acinar architecture was destroyed with focal acinar cell necrosis. Inset: on day 42 after the last ip injection of arginine (on day 54 after the first arginine injection), acinar tissues were replaced by fatty tissue. (Hematoxylin and eosin staining, original magnification ×25).

Subcutaneous (sc) or intraperitoneal (ip) injections of supramaximal stimulatory doses of Cn (20–50 *μ*g/kg body weight) in rats induce a significant increase in serum amylase activity and histological evidence of acute interstitial pancreatitis. Remarkable interstitial edema and cytoplasmic vacuoles in acinar cells are the earliest histological alterations. Cellular infiltration is prominent at 9–12 h after the first Cn injection. However, these histological changes almost completely disappear after 24 h [9]. Intraperitoneal injections of 20 *μ*g/kg body weight Cn in rats, 4 times at hourly intervals, also induce acute pancreatitis. Although recurrent attacks of acute pancreatitis are supposed to be a possible mechanism of CP [10], repeated attacks of Cn-induced pancreatitis for 5 times at weekly intervals were unable to induce CP in rats [11]. Yamaguchi et al. [11] revealed that repetitive injections of Cn cause mild damage of the pancreas parenchyma with inflammatory cell infiltration on day 29 after the first IP Cn injection (1 day after the last Cn). On day 7 after the last Cn injection (35 days after the first Cn injection), however, the epithelium of the pancreatic ductules has recovered from damage, and the pancreas appeared histologically normal (Figure 1(a)).

### 2.2. Arginine-Induced Pancreatitis

Tani et al. [12] have demonstrated that a single ip injection of excessive dose of L-arginine (500 mg/100 g body weight) induces the biological and histological characteristics of acute necrotizing pancreatitis in rats. Histological examination revealed degenerative changes of intracellular organelles and nuclei of acinar cells. The extent and severity of necrotic changes of pancreatic exocrine tissue with inflammatory cell infiltration were maximal at 72 h. Pancreatic acinar cells began to regenerate within 7 days, and pancreatic architecture appeared almost normal after 14 days [12]. Delaney et al. [13] have reported that a single ip injection of 500 mg/100 g body weight of L-arginine followed by 3 injections of 250 mg/100 g body weight over 10 days caused up to 90% acinar destruction with adipose tissue replacement and undamaged ductal, vascular, and islet cells. These changes were present 6 months after injection. Although this procedure is proposed as a new, simple, and reproducible method of inducing chronic pancreatic damage in the rat [13], histological features are completely different from those of CP in humans. Fibrosis is not found, but the destroyed acinar cells were completely replaced by adipose tissue. Weaver et al. [14] have shown that daily ip injections of slightly low dose of arginine (350 mg/100 g body weight) for 4 weeks caused progressive degeneration of the pancreas, and only isolated single acinar cells were seen within a fibrous connective tissue matrix contiguous with ducts, blood vessels, intrapancreatic nerves, and islets. Yamaguchi et al. [11] have demonstrated that ip injections of 500 mg/100 g body weight of L-arginine, 5 times at 3-day intervals destroyed acinar architecture with focal acinar cell necrosis. However, on day 7 after the last arginine injection (on day 22 after the first arginine injection), almost all pancreatic parenchyma are replaced by fatty tissue (Figure 1(b)). Repetitive ip injections of L-arginine appear to produce CP similar to that in humans, but histological appearance is different from that in human CP. In this model, fibrotic tissues are progressively replaced with adipose tissue with time.

### 2.3. Dibutyltin Dichloride-Induced Pancreatitis

Merkord and Hennighausen [15] have shown that a single intravenous (iv) or ip injection of dibutyltin dichloride (DBTC) causes interstitial pancreatitis, enlargement of diameter, and destruction of epithelial cells of the bile ducts depending on the dose of DBTC (1, 4, or 6 mg/kg body weight), the route of administration, and the time after treatment. Single ip injection of DBTC at a dose of 6 mg/kg body weight [16] or 8 mg/kg body weight [17] induced acute interstitial pancreatitis in rats resulting from toxic necrosis of the biliopancreatic duct epithelium and obstructing plugs in the distal common bile duct. Extensive infiltration of the pancreatic interstitium with mononuclear cells was observed after 7 days followed by the development of fibrosis. Twenty-eight days after administration of DBTC, one-third of the rats showed periductal and interstitial fibrosis as well as an active inflammatory process in the pancreas **[**16]. An active inflammatory process was seen even after 2 months [17]. The presence of chronic inflammatory lesions characterized by the destruction of exocrine parenchyma and fibrosis, and in the later stages the endocrine parenchyma, indicates CP [17, 18]. However, Merkord et al. [16] have revealed that only one-third of the treated rats show a tendency to a chronic course, when the obstruction of the duct and cholestasis persist. Thus, this model cannot be used as an experimental model of CP because of the uncertainty of reproducibility and the presence of jaundice.

## 3. Surgical Model of Chronic Pancreatitis

### 3.1. Sodium Taurocholate-Induced Pancreatitis

**Figure 2:**
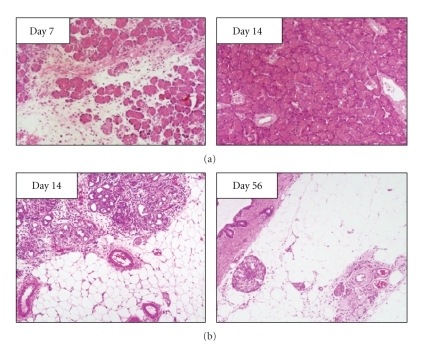
Representative photomicrographs of the pancreas after intraductal infusion of 40 *μ*L/100 g body weight of 3% NaTc solution (a) or after intraductal infusion of 50 *μ*L oleic acid (b). (a-left) On day 7 after intraductal infusion of NaTc, acinar architecture was partially destroyed, and infiltration of inflammatory cells was observed. (a-right) On day 14 after intraductal infusion of NaTc, the pancreas recovered with normal histological features (Hematoxylin and eosin staining, original magnification ×25). (b-left) On day 14 after intraductal infusion of oleic acid, acinar architecture was partially destroyed and replaced by fatty tissue. Tubular complexes were surrounded by fatty tissue. (b-right) On day 56 after intraductal infusion of oleic acid, almost all pancreatic parenchymal tissues were replaced by fatty tissue, and only islet cells were observed (Hematoxylin and eosin staining, original magnification ×25).

Aho et al. [19] have demonstrated that retrograde infusion of sodium taurocholate (NaTc) into the pancreatic duct system of the rat causes acute hemorrhagic pancreatitis. The pancreatic lesions are immediate and characterized by interstitial edema, extensive necrotic changes of the acinar cells, and hemorrhages during the first 24 h after the injection. The mortality increased depending on the amount and concentration of the injected NaTc. Marked acinar atrophy and pancreatic fibrosis are seen in rats surviving 72 h [19]. In contrast, Yamaguchi et al. [11] have demonstrated that pancreatitis induced by intraductal infusion of 40 *μ*L/100 g body weight of 3% NaTc solution is reversible (Figure 2(a)). Partially destructed acinar architecture and infiltration of inflammatory cells were seen from day 3 to day 7 after intraductal infusion of NaTc (Figure 2(a), left). On day 14, however, the structure of the pancreatic tissue returned to almost normal (Figure 2(a), right).

 In this model, rats that were infused with a large amount or high concentration of NaTc solution died, whereas rats infused with a small amount of NaTc solution at a low concentration recovered within 14 days. It is difficult to control the amount and the concentration of NaTc solution to induce pancreatic fibrosis.

### 3.2. Oleic Acid-Induced Pancreatitis

Retrograde infusion of oleic acid [11, 20–23], viscous solution of zein [24], mixture of zein-oleic acid, or viscous solution consisting of zein-oleic acid-linoleic acid [25, 26] (50 *μ*L/100 g body weight) into rat pancreatic duct causes severe pancreatic atrophy with irregular fibrosis and fat replacement over a period of 6 months. Indeed, intraductal infusion of 50 *μ*L/rat of oleic acid destroyed acinar cells and pancreatic ductulus epithelia and induced infiltration of inflammatory cells on day 3 [22]. Inflammatory cell infiltration further increased until day 14 (Figure 2(b), left), but almost all pancreatic parenchymal cells were replaced by fatty tissues by day 56 (Figure 2(b), right). Although these animals also developed malabsorption of fat and bentiromide and showed retardation of body weight in spite of normal food intake [20, 25, 26]; pancreatic fibrosis was rarely seen except fatty tissues. Kataoka et al. [26] have proposed that obstructive mechanism in the small ducts might have played an important role in the genesis and development of CP in this model. Retrograde intraductal infusion of these substances produced a reproducible and long-lasting atrophy of the exocrine pancreas with fatty replacement. These results suggest that viscous substance in the duct or disturbance of pancreatic fluid flow plays an important role in the development of persistent damage of the pancreas.

However, these models of pancreatitis appear quite different from CP in humans. Repetitive injections of Cn or intraductal infusion of NaTc caused transient pancreatitis, and the pancreatic injury returned histologically normal within 14 days. On the other hand, repetitive injection of arginine or intraductal infusion of oleic acid induced persistent damage of the pancreas, progressing to pancreatic insufficiency with replacement of almost all pancreatic parenchymal tissues by fatty tissues without fibrosis. Thus, histopathological changes in the pancreas of these rats are different from those observed in human CP.

## 4. Congestion of Pancreatic Fluid Flow

**Figure 3:**
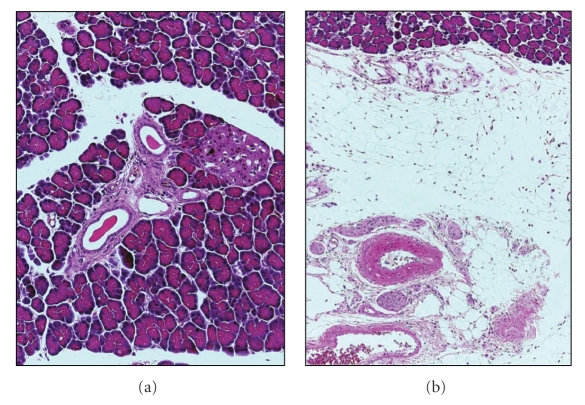
Representative photomicrographs of the pancreas after complete ligation of the main pancreatic duct. Common bile duct was ligated proximal to the pancreas near the liver, and a cannula was inserted above the ligature, and the bile was introduced into the duodenum via cannula so as to prevent obstructive jaundice. The biliopancreatic duct was completely ligated at its entrance into the duodenum. (a) On day 7 after the ligation, pancreatic fibrosis was observed only around the slightly dilated duct. (b) On day 14 after the ligation, pancreatic parenchymal tissues were replaced by fatty tissue without fibrosis. (Hematoxylin and eosin staining, original magnification ×25).

In the initial lesions of human CP, protein plugs are found in small pancreatic ducts [7, 27]. In addition, several studies have shown that the viscosity of pancreatic juice is increased in patients with CP [28, 29], and that pancreatic ductal pressure in patients with CP is significantly higher than that in control subjects [30]. Surgical decompression of the pancreatic duct [31], endoscopic ductal drainage [29, 32], or removal of intraductal stones by extracorporeal shockwave lithotripsy (ESWL) improves the clinical outcome of patients with CP [33]. Taken together, congestion of pancreatic fluid flow or pancreatic ductal hyperpressure (PDH) seems to contribute to the pathogenesis of CP. Indeed, obstruction of the main pancreatic duct by tumors, strictures, or anatomic variants such as pancreas divisum is another possible cause of CP [34]. Although these previous studies have demonstrated that PDH contributes to the pathogenesis of chronic pancreatitis, complete ligation of the pancreatic duct causes pancreatic atrophy and fatty degradation rather than fibrosis (Figure 3).

### 4.1. Combination of Transient Stasis of Pancreatic Juice Flow and Mild Pancreatic Duct Injury

**Figure 4:**
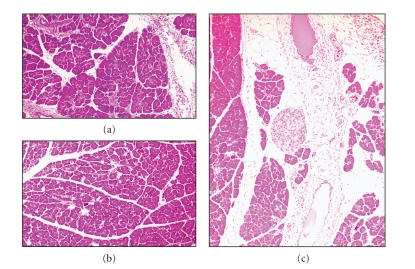
Representative photomicrographs of the pancreas after intraductal infusion of 40 *μ*L/100 g body weight of 0.1% NaTc solution (a), after intraductal infusion of 40 *μ*L of 0.01% agarose (b), or after intraductal infusion of a mixture solution of 0.1% NaTc and 0.01% agarose at amount of 40 *μ*L/100 g body weight (c). (a) On day 14 after intraductal infusion of 40 *μ*L/100 g body weight of 0.1% NaTc solution, the pancreas was histologically normal. (b) On day 14 after intraductal infusion of 40 *μ*L/100 g body weight of 0.01% agarose, the pancreas appeared almost normal. (c) On day 14 after intraductal infusion of a mixture of 0.01% agarose and 0.1% NaTc solution at an amount of 40 *μ*L/100 g body weight, acinar architecture was partially destroyed and replaced by fatty tissues. Dilated pancreatic duct with focal acinar cell necrosis and inflammatory cell infiltration were seen. (Hematoxylin and eosin staining, original magnification ×25).

Yamaguchi et al. [11] have reported that pancreatic injury induced by a retrograde intraductal infusion of 40 *μ*L/100 g body weight of 3.0% NaTc solution was transient and returned to normal within 14 days (Figure 2(a)). On the other hand, previous studies have suggested the importance of the disturbance of pancreatic fluid flow for the development of persistent damage of the pancreas [11, 20–26]. We, therefore, infused a mixture solution of low-concentration NaTc (0.1%) and viscous agarose solution (0.01%) to induce mild injury of the pancreatic duct and a transient stasis of pancreatic fluid flow. Partial destruction of acinar architecture, infiltration of inflammatory cells, and dilatation of pancreatic ducts were seen on day 7 in rats after intraductal infusion of 40 *μ*L/100 g body weight of 0.1% NaTc solution but returned almost normal structure on day 14 (Figure 4(a)). Mild damage of the parenchymal tissue with inflammatory cell infiltration that was observed on day 7 after intraductal infusion of 40 *μ*L/100 g body weight of 0.01% agarose completely recovered on day 14 (Figure 4(b)). However, intraductal infusion of a mixture 0.01% agarose and 0.1% NaTc solution at an amount of 40 *μ*L/100 g body weight destroyed acinar architecture with focal acinar cell necrosis and inflammatory cell infiltration (Figure 4(c)). Even on day 56, pancreatic interstitial fibrosis, acinar damage, and dilated pancreatic ducts were seen. Acinar cell necrosis was surrounded by fatty tissue. This model of pancreatitis clearly revealed that both stasis of pancreatic fluid flow and ductal injury are necessary for the development of persistent injury of the pancreas. Transient stasis of pancreatic fluid flow and minimum injury of the pancreatic duct act synergistically to cause progressive pancreatic injury, although one alone is insufficient to cause persistent pancreatic injury.

This new model of pancreatitis revealed the importance of a combination of the disturbance of pancreatic fluid flow and the injury of pancreatic duct to induce persistent and irreversible changes of the pancreas. However, the resulting histological features are different from those of CP in human because pancreatic fibrosis is rarely seen and pancreatic parenchyma is replaced by fatty tissue.

## 5. Pancreatic Ductal Hypertension

Although PDH contributes to the pathogenesis of CP, complete obstruction of the pancreatic duct results in pancreatic atrophy and fatty degradation rather than fibrosis (Figure 3). Yamamoto et al. [35] developed an animal model with PDH and demonstrated that continuous PDH plays an important role in the onset and development of CP in rats. 

**Figure 5:**
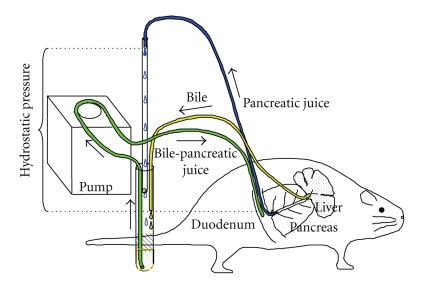
Schematic diagram of the experimental setup. The height from the level of the ampulla Vater to the free end of the pancreatic cannula represents the hydrostatic pressure to the pancreas.

Common bile duct was ligated proximal to the pancreas near the liver, and a cannula was inserted above the ligature to collect pure bile. Another cannula was inserted into the biliopancreatic duct through the ampulla of Vater to collect pure pancreatic juice. Pancreatic juice and bile were continuously returned to the duodenum by a servomechanism. An additional cannula was inserted into the duodenum to return bilopancreatic juice (Figure 5). During the recovery and experimental periods, the rat was placed in a modified Bollman-type restraint cage and had full access to food and water ad libitum. 

The height from the level of the ampulla Vater to the free end of the pancreatic cannula represents the hydrostatic pressure to the pancreas, which was increased to 25 cm by vertically raising the free end of the pancreatic cannula from the pancreas (Figure 5). The hydrostatic pressure was raised 5 cm each day from 25 to 35 cm but not above 35 cm to prevent acute pancreatic damage. Pancreatic ductal pressure was controlled to obtain 25% pancreatic fluid flow of the control rats that were subjected to the same operative procedures, but the free end of the pancreatic cannula was maintained at 4-5 cm below the pancreas to maintain pancreatic fluid flow. 

Rats in the PDH group consumed nearly the same amount of food as those in the control group during the experimental period, but they failed to gain body weight and evacuated muddy or soft feces, indicating maldigestion probably due to reduced pancreatic enzyme secretion. Indeed, amylase and lipase activity in pancreatic fluid progressively decreased after induction of PDH. Pancreatic exocrine function evaluated by iv secretin stimulation on day 14 revealed pancreatic insufficiency in the PDH rats. However, nonenzymatic protein concentration in pancreatic fluid gradually increased during continuous PDH after the initial drop. 

**Figure 6:**
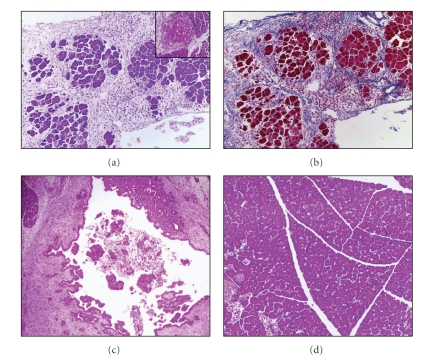
Representative light microscopic appearance of the pancreas in PDH and control groups on day 14 after the induction of PDH. (a) Pancreatic fibrosis was mainly distributed in the interlobular area with a nodular and lobular pattern. Marked infiltration of inflammatory cells was observed mainly in the interlobular area. Inset: representative light microscopic picture of focal fat necrosis. (b) Azan-stained section showed replacement of normal tissue by fatty and connective tissue. (c) Plug formation in the main pancreatic duct. (d) Minimal histological changes were observed in the control group on day 14. (Hematoxylin and eosin staining, original magnification ×25).

Pancreatic fibrosis observed mainly in the intralobular area and especially in the periductal area on day 7 after the induction of PDH further progressed and was markedly distributed in the interlobular or perilobular and intralobular areas on day 14 (Figures 6(a) and 6(b)). In addition, marked inflammatory cell infiltration, predominantly lymphocytes, was observed in interlobular and intralobular areas. Focal fat necrosis (Figure 6(a)-inset) and plug formation in the main pancreatic duct (Figure 6(c)) were also observed on day 14. These histopathological changes are reliable, reproducible, and closely resemble human CP. The significantly increased concentration of mucoprotein in pancreatic fluid might have increased the viscosity of pancreatic fluid and contributed to the formation of protein plugs. In control rats underwent similar operative procedure but without PDH, the pancreas showed only minimal histological changes on day 14 (Figure 6(d)).

**Figure 7:**
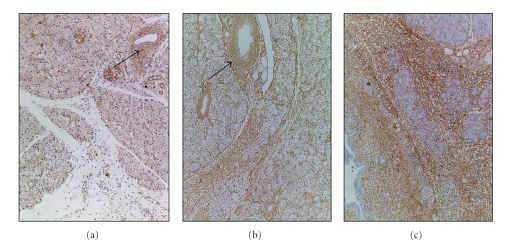
Immunohistochemistry for collagen type I (a), collagen type III (b), and fibronectin (c) in the pancreas on day 14 after the induction of PDH. (a) Collagen type I was markedly distributed around the pancreatic duct but was slightly distributed in interlobular and intralobular areas. (b, c) Collagen type III (b) and fibronectin (c) were markedly distributed in interlobular and intralobular areas and around the pancreatic duct ×100.

**Figure 8:**
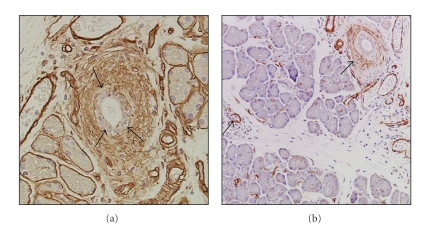
Immunohistochemistry for collagen type IV (a) and *α*-smooth muscle actin- (*α*-SMA-) positive cells in the pancreas on day 14 after the induction of PDH. (a) Type IV collagen deposition was observed as irregular and discontinuous lines along the basement membrane of the ducts. In addition, broad deposition of type IV collagen was observed around the ducts. (b) *α*-SMA-positive cells were detected in the periductal, periacinar, and interstitial areas. Original magnification ×200.

Immunoreactivity for type I collagen was markedly detected around pancreatic ducts and slightly detected in interlobular and intralobular areas (Figure 7(a)). On the other hand, collagen type III (Figure 7(b)) and fibronectin (Figure 7(c)) were markedly detected around the pancreatic ducts and in interlobular and intralobular areas. Type IV collagen deposition was observed as irregular and discontinuous lines along the basement membrane (BM) of the ducts (Figure 8(a)), as have been observed in arginine- and oleic acid-induced pancreatitis [11, 22] and in human CP [36–38]. In addition, broad deposition of type IV collagen was observed around the ducts (Figure 8(a)). On the other hand, in the control rats, type IV collagen deposition was observed as continuous lines along the BM of the ducts [35], as has been found in the normal rat [11, 22] and normal human pancreas [36, 37]. 

In the PDH rats, *α*-smooth muscle actin- (*α*-SMA-) positive cells, activated pancreatic stellate cells (PSCs), were observed in periductal, periacinar, and interlobular areas (Figure 8(b)). However, in the control group *α*-SMA-positive cells were rarely found except in the vessel walls [35]. PDH markedly increased the expression level of TGF-*β* transcript and was associated with a progressive and significant increase in the proportion of the Azan-Mallory blue-stained fibrotic area (Figure 6(b)). Thus, PDH plays an important role in the formation of pancreatic fibrosis.

## 6. Conclusion

Although repetitive injections of arginine or intradauctal infusion of oleic acid or a mixture of agarose and NaTc caused persistent pancreatic injury, the damaged tissue was replaced by fatty tissue without fibrosis. Continuous PDH caused diffuse interlobular and intralobular fibrosis resembling human CP and resulted in pancreatic exocrine function with reduced body weight gain and muddy or soft feces in the presence of unaltered food intake. Therefore, the PDH model seems suitable for the investigation of the mechanism of CP. However, there are several limitations of this animal model; surgical and maintenance skills and equipments are required for the present experimental model. In addition, it is difficult to maintain this animal model for more than 3 weeks.
